# *trans*-Fatty acids promote p53-dependent apoptosis triggered by cisplatin-induced DNA interstrand crosslinks via the Nox-RIP1-ASK1-MAPK pathway

**DOI:** 10.1038/s41598-021-89506-8

**Published:** 2021-05-14

**Authors:** Yusuke Hirata, Miki Takahashi, Yuto Yamada, Ryosuke Matsui, Aya Inoue, Ryo Ashida, Takuya Noguchi, Atsushi Matsuzawa

**Affiliations:** Laboratory of Health Chemistry, Graduate School of Pharmaceutical Sciences, Tohoku University, 6-3 Aoba, Aramaki, Aoba-ku, Sendai, Miyagi 980-8578 Japan

**Keywords:** Kinases, Lipids, Risk factors, Cell death, Cell signalling

## Abstract

*trans*-Fatty acids (TFAs) are food-derived fatty acids associated with various diseases including cardiovascular diseases. However, the underlying etiology is poorly understood. Here, we show a pro-apoptotic mechanism of TFAs such as elaidic acid (EA), in response to DNA interstrand crosslinks (ICLs) induced by cisplatin (CDDP). We previously reported that TFAs promote apoptosis induced by doxorubicin (Dox), a double strand break (DSB)-inducing agent, via a non-canonical apoptotic pathway independent of tumor suppressor p53 and apoptosis signal-regulating kinase (ASK1), a reactive oxygen species (ROS)-responsive kinase. However, here we found that in the case of CDDP-induced apoptosis, EA-mediated pro-apoptotic action was reversed by knockout of either p53 or ASK1, despite no increase in p53 apoptotic activity. Upon CDDP treatment, EA predominantly enhanced ROS generation, ASK1-p38/c-Jun N-terminal kinase (JNK) mitogen-activated protein kinase (MAPK) pathway activation, and ultimately cell death, all of which were suppressed either by co-treatment of the NADPH oxidase (Nox) inhibitor Apocynin, or by knocking out its regulatory protein, receptor-interacting protein 1 (RIP1). These results demonstrate that in response to CDDP ICLs, TFAs promote p53-dependent apoptosis through the enhancement of the Nox-RIP1-ASK1-MAPK pathway activation, providing insight into the diverse pathogenetic mechanisms of TFAs according to the types of DNA damage.

## Introduction

*trans*-fatty acids (TFAs) are unsaturated fatty acids that contain at least one *trans* carbon–carbon double bond. In the human body, while *cis*-fatty acids (CFAs) containing only *cis* carbon–carbon double bonds are enzymatically synthesized and essential for a variety of biological functions, TFAs are not synthesized and thus obtained from dietary sources^1^. Based on the difference in source derivation, TFAs are categorized into two groups: industrial and ruminant. Industrial TFAs, such as elaidic acid (EA, C18:1 *t*9) and linoelaidic acid (LEA, C18:2 *t*9,*t*12), are produced as byproducts in food manufacturing processes (e.g. partial hydrogenation of edible oils), and are present in various processed foods including margarine, shortening and snacks^1^. On the other hand, ruminant TFAs, such as *trans*-vaccenic acid (tVA, C18:1 *t*11), are produced during enzymatic biohydrogenation of CFAs by rumen microbes in cows and sheep, and are present in meat and dairy products^1^. Accumulating epidemiological and in vivo studies have linked TFAs, particularly industrial TFAs, with various diseases such as metabolic syndrome, inflammatory diseases, neurodegenerative diseases (NDs), and cardiovascular diseases (CVDs)^2–5^. However, the underlying pathological mechanisms remain elusive owing to the lack of molecular-level studies.

DNA is damaged by a wide variety of endogenous and environmental stresses, such as DNA replication, UV, reactive oxygen species (ROS), and anti-cancer drugs (e.g. doxorubicin (Dox) and cisplatin (*cis*-diamminedichloro-platinum (II): CDDP), which lead to various types of DNA lesions^6,7^. Since DNA lesions give rise to genomic instability and gene mutations that contribute to numerous diseases including cancer, according to the types and extent of DNA lesions, cells induce appropriate cellular responses, such as DNA repair, cell cycle arrest, senescence, and cell death, which are known as DNA damage responses (DDRs)^8^. When DNA is damaged beyond repair, cells undergo programmed cell death including apoptosis, which has been closely linked to pathogenesis of TFA-related diseases, such as atherosclerosis^9,10^ and NDs^11^. Our recent study revealed a pro-apoptotic function of TFAs in response to DNA damage induced by Dox, a DNA topoisomerase II (Top II) inhibitor that efficiently induces DNA strand breaks, mainly double strand breaks (DSBs), via forming Top II-DNA covalent complexes^12^. p53 is a well-known tumor suppressor protein that is induced by DNA damage, and serves as a master regulator in the DDR by transcriptional upregulation of target genes such as p21, Bcl-2-associated X protein (Bax), and p53-upregulated modulator of apoptosis (Puma)^13^. However, we showed that when cells are treated with Dox, TFAs facilitate apoptosis independently of p53, but rather in a manner dependent on the mitochondrial c-Jun N-terminal kinase (JNK)-Sab (also named as SH3 domain binding protein 5, SH3BP5)-ROS axis. Sab is an adaptor protein that is localized at the mitochondrial outer membrane, and recruits activated JNK in response to various stresses including DNA damage, thereby triggering mutual enhancement of mitochondrial ROS (mitoROS) generation and JNK activation^14^. TFAs augment Dox-induced mitoROS generation via the mitochondrial JNK-Sab pathway, resulting in JNK/p38 mitogen-activated protein kinase (MAPK) hyperactivation and ultimately apoptosis. Although we previously demonstrated that TFAs also promote apoptosis in response to CDDP, an alkylating agent that causes DNA interstrand crosslinks (ICLs) blocking DNA replication and RNA transcription, it remains to be elucidated whether in this case TFAs elicit pro-apoptotic signals in a similar mechanism as in the case of Dox.

In this study, we found a novel TFA-mediated pro-apoptotic signaling that is specifically induced by CDDP, but not by Dox. We showed that industrial TFAs, such as EA and LEA, promoted CDDP-induced apoptosis in a p53-dependent manner. This effect was not observed in the corresponding *cis* isomer of TFAs and only marginally in palmitic acid (PA, 16:0), one of the major saturated fatty acids (SFAs). EA primarily enhanced NADPH oxidase (Nox)-dependent ROS production during CDDP-induced DNA damage and promoted subsequent activation of the apoptosis signal-regulating kinase (ASK1)-p38/JNK MAPK pathway, and ultimately apoptosis. These results demonstrate diverse molecular mechanisms of TFA toxicity in response to DNA damage, which will provide a comprehensive understanding of the essential molecular targets of TFAs and the pathogenesis of TFA-related diseases.

## Results

### Major industrial TFAs promote CDDP-induced apoptosis

**Figure 1:**
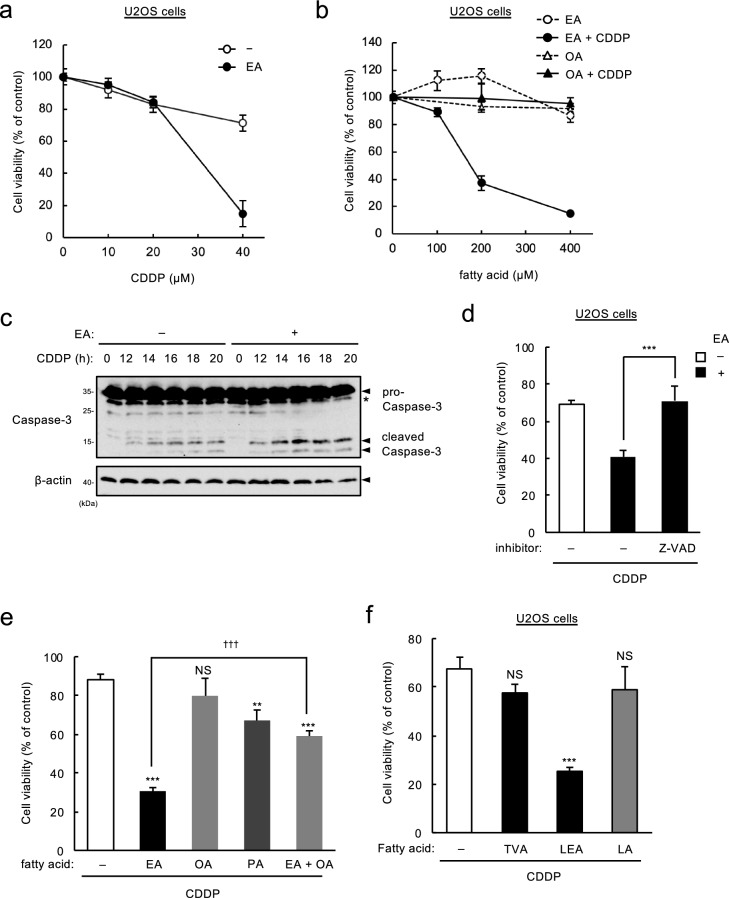
Major industrial TFAs promote CDDP-induced apoptosis. (**a**) U2OS cells were pretreated with or without 200 μM EA for 12 h, and then stimulated with various concentrations of CDDP for 24 h, subjected to cell viability assay. Data shown are the mean ± SD (n = 3). (**b**) U2OS cells were pretreated with the indicated concentrations of OA or EA for 12 h, and then stimulated with or without 40 μM CDDP for 24 h, subjected to cell viability assay. Data shown are the mean ± SD (n = 3). (**c**) U2OS cells were pretreated with or without 200 µM EA for 12 h, and then stimulated with 40 μM CDDP for the indicated time periods. Cell lysates were subjected to immunoblotting with the indicated antibodies. *, non-specific band. (**d**) U2OS cells were pretreated with or without 200 µM EA for 11.5 h, treated with the pan-caspase inhibitor z-VAD-fmk for 0.5 h, and followed by stimulation with 40 μM CDDP for 24 h, subjected to cell viability assay. Data shown are the mean ± SD (n = 3). (**e** and **f**) U2OS cells were pretreated with the indicated fatty acids at 200 μM for 12 h, and then stimulated with 40 μM CDDP for 24 h, subjected to cell viability assay. Data shown are the mean ± SD. NS, not significant; **p < 0.01; ***p < 0.001 (vs. cells without any fatty acid); ^†††^*p* < 0.001.

To evaluate the effect of TFAs on CDDP-induced cell death, we first examined whether EA, the most abundant TFA in processed foods^1^, enhances cell death induced by CDDP treatment in U2OS (human osteosarcoma) cells. As shown in Fig. 1a, when cells were treated with 40 μM CDDP, pretreatment with EA markedly decreased cell viability. EA itself was not cytotoxic up to 400 µM, but its pretreatment substantially reduced cell viability in response to CDDP, whereas that of OA, as a *cis* isomer of EA, did not (Fig. 1b). This EA-specific effect was also observed in other cell lines, such as RAW264.7 (mouse macrophage-like) cells and TIG-3 (human normal fibroblast) cells (see Supplementary Fig. S1 online). Immunoblot analysis showed that EA enhanced CDDP-induced caspase-3 cleavage, a typical apoptosis marker (Fig. 1c). In addition, EA-mediated decrease in cell viability was completely suppressed by co-treatment with a pan-caspase inhibitor z-VAD-fmk (Fig. 1d). These data suggest that EA, but not OA, promotes apoptosis induced by CDDP. Physiological properties of TFAs resemble to SFAs compared to CFAs^2^. We thus investigated whether palmitic acid (PA, C16:0), a typical SFA, promotes CDDP-induced cell death. PA pretreatment significantly promoted CDDP-induced cell death, but to much lesser extent than EA pretreatment (Fig. 1e), indicating that EA has much higher potency than SFAs in enhancing CDDP-induced apoptosis. We previously showed that in the case of Dox-induced cell death, EA-mediated pro-apoptotic effect was counteracted by simultaneous pretreatment of OA, and that LEA, another major TFA in processed foods, but not TVA, the most abundant TFA in ruminant-derived foods, also potently promoted Dox-induced cell death^12^. We consistently found that OA clearly suppressed the increase in CDDP-induced cell death mediated by EA (Fig. 1e). Furthermore, pretreatment of LEA, but not TVA or linoleic acid (LA, C18:2 *c*9,*c*12) as a *cis* isomer of LEA, dramatically augmented cell death induced by CDDP (Fig. 1f). These results indicate that major industrial TFAs, EA and LEA, efficiently promote CDDP-induced apoptosis, similarly as in the case of Dox-induced apoptosis.

### EA promotes CDDP-induced apoptosis in a p53-dependent manner without enhancement of its expression or apoptotic activity

**Figure 2:**
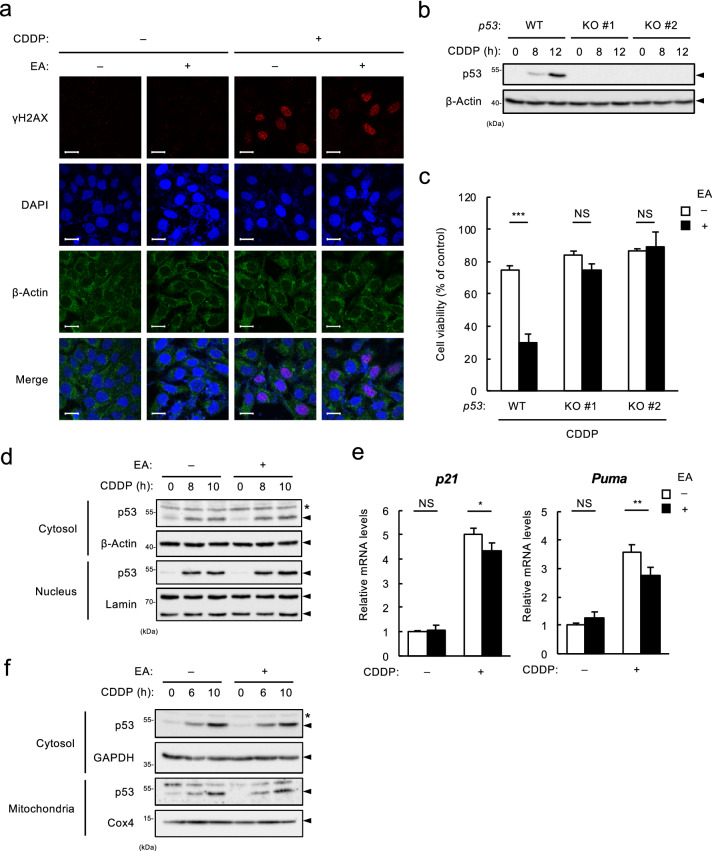
EA promotes CDDP-induced apoptosis in a p53-dependent manner without increase in its expression and activity. (**a**) U2OS cells were pretreated with or without 200 µM EA for 12 h, and then stimulated with 40 μM CDDP for 6 h, subjected to immunocytochemistry using antibodies against γ-H2AX and β-actin. Scale bar, 20 µm. (**b**) *p53* WT and KO U2OS cells were treated with 40 μM CDDP for the indicated time periods. Cell lysates were subjected to immunoblotting with the indicated antibodies. (**c**) *p53* WT and KO U2OS cells were pretreated with or without 200 μM EA for 12 h, and then treated with 40 μM CDDP for 24 h, subjected to cell viability assay. Data shown are the mean ± SD (n = 3). (**d**) U2OS cells were pretreated with or without 200 µM EA for 12 h, and then stimulated with 40 μM CDDP for the indicated time periods. Cytoplasmic and nuclear extracts obtained from the cell lysates were subjected to immunoblotting with the indicated antibodies. *, non-specific band. (**e**) U2OS cells were pretreated with or without 200 µM EA for 12 h, and then stimulated with 40 μM CDDP for 16 h, subjected to qRT-PCR analysis. Relative mRNA levels of *p21* and *Puma* are shown as mean ± SD (n = 3). (**f**) U2OS cells were pretreated with or without 200 µM EA for 12 h, and then stimulated with 40 μM CDDP for the indicated time periods. Cytoplasmic and mitochondrial extracts obtained from the cell lysates were subjected to immunoblotting with the indicated antibodies. *, non-specific band.

To elucidate the mechanism by which EA promotes CDDP-induced cell death, we first examined the involvement of p53, a transcription factor that plays the most prominent role in DDR^8^. Before analysis, we confirmed that EA does not affect CDDP-induced histone 2AX phosphorylation (known as γ-H2AX), one of the most common DNA damage marker, indicating that EA has no apparent effect on DNA damage accumulation induced by CDDP (Fig. 2a and Supplementary Fig. S2 online). We then established *p53* knockout (KO) U2OS cell lines using the CRISPR/Cas9 system, which completely lack p53 expression either in the absence or presence of CDDP (Fig. 2b). We found that in *p53* KO cells, EA-dependent increase in CDDP-induced cell death was almost completely abolished (Fig. 2c), while that in Dox-induced cell death was apparently not (see Supplementary Fig. S3 online), in line with our previous report^12^. These data suggest that EA promotes CDDP-induced cell death in a p53-dependent manner, unlike its p53-independent promoting effect on Dox-induced cell death^12^. We then investigated whether EA augments CDDP-induced p53 activation. However, we found that EA potentiated neither p53 upregulation nor its translocation to the nucleus induced by CDDP (Fig. 2d). Moreover, EA did not enhance, but rather slightly diminished CDDP-induced transcriptional induction of p53 target genes, including *p21* and *Puma*, for unknown reasons (Fig. 2e). Upon DNA damage, a subset of p53 localizes to the mitochondria, facilitates mitochondrial outer membrane permeabilization (MOMP), and thereby promotes apoptosis^15^. We nevertheless observed that CDDP-induced p53 mitochondrial translocation was not affected in the presence or absence of EA (Fig. 2f). Taken together, these results suggest that EA promotes CDDP-induced apoptosis in a p53-dependent manner without enhancement of its expression or apoptotic activity. Although p53 is required for EA-mediated enhancement of CDDP-induced cell death, the effective target of EA is not p53. Probably, other essential targets exist.

### Pro-apoptotic action of EA depends on hyperactivation of the ASK1-p38/JNK MAPK pathway

**Figure 3:**
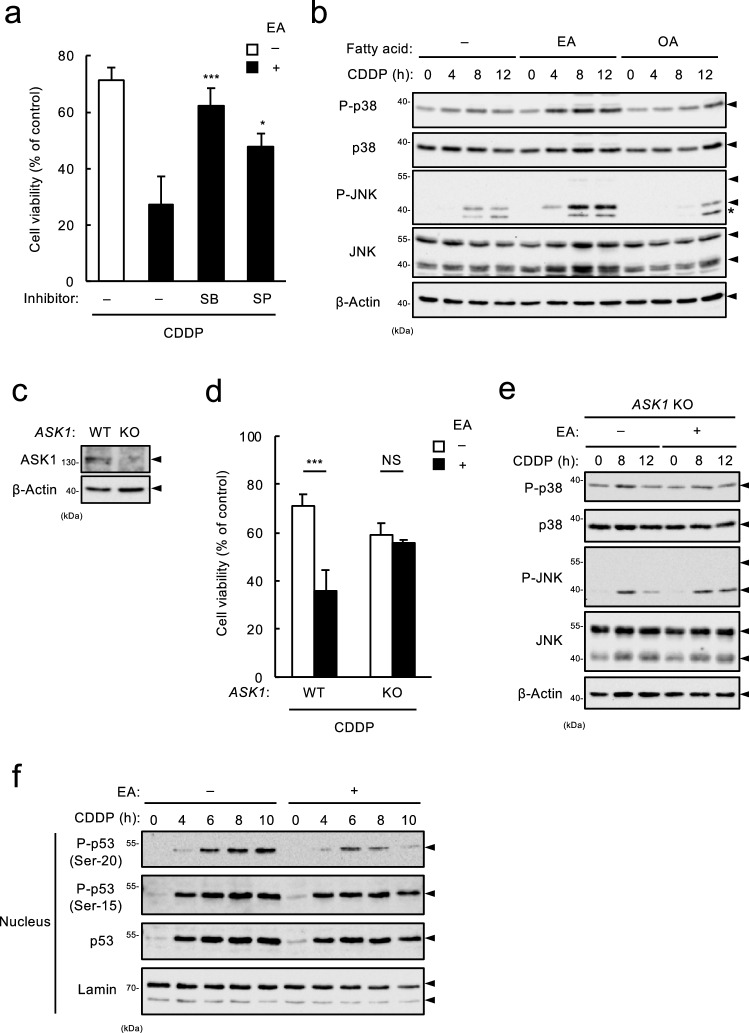
Pro-apoptotic action of EA depends on hyperactivation of the ASK1-p38/JNK MAP kinase pathway. (**a**) U2OS cells were pretreated with 200 μM EA for 11.5 h, treated with either p38 inhibitor SB203580 (SB, 5 μM) or JNK inhibitor SP600125 (SP, 5 μM) for 0.5 h, and then stimulated with 40 μM CDDP for 24 h, subjected to cell viability assay. Data shown are the mean ± SD (n = 3). *p < 0.05; ***p < 0.001 (vs. cells pre-treated with EA in the absence of any inhibitor). (**b**) U2OS cells were pretreated with 200 µM EA or OA for 12 h, and then stimulated with 40 μM CDDP for the indicated time periods. Cell lysates were subjected to immunoblotting with the indicated antibodies. *, non-specific band. (**c**) *ASK1* WT and KO U2OS cells were lysed and subjected to immunoblotting with the antibodies against ASK1 and β-actin. (**d**) *ASK1* WT and KO U2OS cells were pretreated with or without 200 μM EA for 12 h, and then treated with 40 μM CDDP for 24 h, subjected to cell viability assay. Data shown are the mean ± SD (n = 3). (**e**) *ASK1* WT and KO U2OS cells were pretreated with or without 200 µM EA for 12 h, and then stimulated with 40 μM CDDP for 0, 8 and 12 h. Cell lysates were subjected to immunoblotting with the indicated antibodies. (**f**) U2OS cells were pretreated with or without 200 µM EA for 12 h, and then stimulated with 40 μM CDDP for the indicated time periods. Nuclear extracts obtained from the cell lysates were subjected to immunoblotting with the indicated antibodies.

We have shown the key roles of stress-responsive p38/JNK MAPK pathway in TFA-mediated promoting effect on apoptosis induced by Dox and extracellular ATP, one of the damage-associated molecular patterns secreted from injured cells^12,16,17^. We therefore examined the involvement of stress-responsive MAPKs in promoting effect by EA on CDDP-induced apoptosis. Cell survival assay revealed that EA-dependent increase in CDDP-induced cell death was significantly suppressed by co-treatment with either p38 inhibitor SB203850 (SB) or JNK inhibitor SP600125 (SP) (Fig. 3a). Moreover, immunoblot analysis showed that CDDP-induced activation of p38 and JNK was clearly increased by pretreatment of EA, but not by that of OA (Fig. 3b), suggesting that EA enhances p38/JNK activation in response to CDDP, thereby promoting cell death. To investigate whether ASK1, a MAP3 kinase upstream of p38/JNK, contributes to the EA-mediated p38/JNK hyperactivation, we established *ASK1* KO U2OS cell lines (Fig. 3c). We found that in *ASK1* KO cells, EA-mediated enhancement of cell death (Fig. 3d) and p38/JNK activation (Fig. 3e) was clearly suppressed, while not in *p53* KO cells (see Supplementary Fig. S4 online). These results suggest that ASK1 is responsible for EA-mediated augmentation of p38/JNK MAPK activation and subsequent p53-dependent cell death during CDDP-induced DNA damage, in contrast with the case of Dox-induced cell death promoted by EA in an ASK1-independent fashion^12^. To confirm the assumption that the EA-mediated p38/JNK hyperactivation does not affect p53 expression or activity (Fig. 2d–f), we monitored p53 phosphorylation status at Ser15 and Ser20, which are known to be phosphorylated by p38 and JNK, respectively, in response to DNA damage, and thereby positively regulate p53 stability and transcriptional activity^18^. As expected, CDDP-induced p53 phosphorylation at Ser15 was comparable with or without EA, and that at Ser20 was even slightly decreased by EA pretreatment (Fig. 3f), which is consistent with a slight decrease in transcriptional induction of p53 target genes (Fig. 2e). This result supports the notion that EA-mediated p38/JNK hyperactivation contributes to the enhancement of p53-dependent apoptosis signaling without upregulation of p53 expression or activity.

### EA augments CDDP-induced cell death by enhancing ROS generation via NADPH oxidase

**Figure 4:**
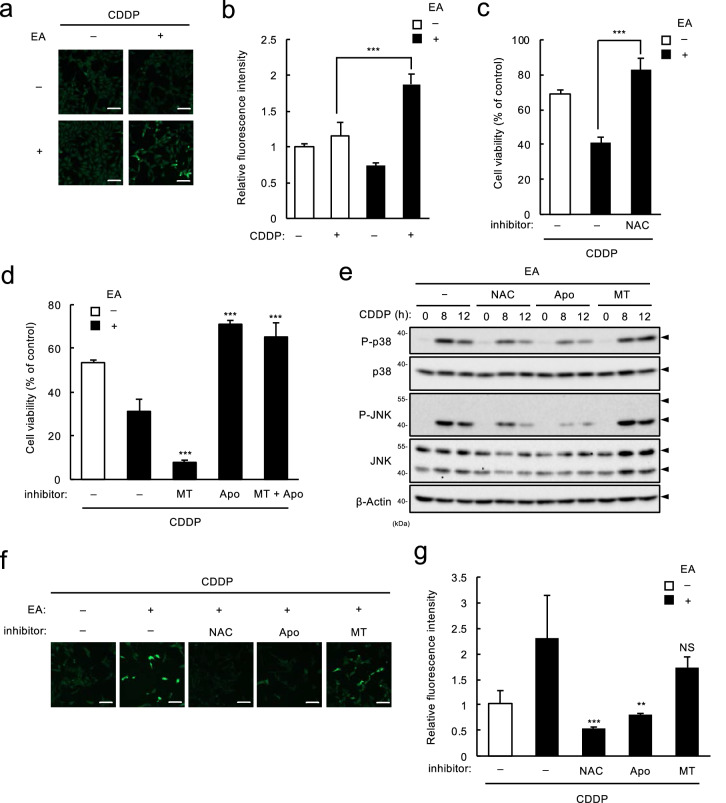
EA promotes CDDP-induced cell death through enhancement of ROS generation by NADPH oxidase. (**a** and **b**) U2OS cells were pretreated 200 µM EA for 12 h, and then stimulated with 40 μM CDDP for 9 h, followed by incorporation of a ROS-sensitive fluorescent probe DCFH-DA for 30 min. Green fluorescence was observed by confocal microscope (**a**), and relative ROS levels were calculated, shown as mean ± SD (n = 3, normalized to the ROS level in the cells without fatty acid and CDDP) (**b**). Scale bar, 100 µm. (**c**) U2OS cells were pretreated with or without 200 µM EA for 11.5 h, treated with antioxidant N-acetylcysteine (NAC, 1 mM) for 0.5 h, and then stimulated with 40 μM CDDP for 24 h, subjected to cell viability assay. Data shown are the mean ± SD (n = 3). (**d**) U2OS cells were pretreated with 200 μM EA for 11.5 h, treated with either mito-Tempo (MT, 10 μM) or Apocynin (Apo, 100 μM) alone or together for 0.5 h, and then stimulated with 40 μM CDDP for 24 h, subjected to cell viability assay. Data shown are the mean ± SD (n = 3). ***p < 0.001 (vs. EA-pretreated cells without any inhibitor). (**e**) U2OS cells were pretreated with 200 μM EA for 11.5 h, treated with either 1 mM NAC, 100 μM Apo and 10 μM MT for 0.5 h, and then stimulated with 40 μM CDDP for the indicated time periods. Cell lysates were subjected to immunoblotting with the indicated antibodies. (**f** and **g**) U2OS cells were pretreated with or without 200 µM EA for 12 h, and then stimulated with 40 μM CDDP for 6 h, followed by incorporation of a ROS-sensitive fluorescent probe DCFH-DA for 30 min. Green fluorescence was observed (**f**) and relative ROS levels were calculated (**g**), shown as mean ± SD (n = 3, normalized to the ROS level in the cells without fatty acid and CDDP). Scale bar, 100 µm. **p < 0.01; ***p < 0.001.

Since ROS is the most potent activator of ASK1^19^, we next investigated whether EA involves ROS induction by CDDP. Intracellular ROS measurement using the fluorescence ROS indicator DCFH-DA showed that CDDP treatment induced a remarkable increase in fluorescence with EA pretreatment, suggesting a crucial role of EA as an enhancer of ROS generation (Fig. 4a,b). To examine the significance of the increase in ROS generation, we next performed cell survival assay with an antioxidant N-acetylcysteine (NAC). As shown in Fig. 4c, NAC co-treatment canceled the EA-dependent enhancement of CDDP-induced cell death, indicating a prominent contribution of the increased ROS generation to the EA-mediated enhancement of CDDP-induced apoptosis. NADPH oxidase (Nox) and mitochondria are the major sources of intracellular ROS generation^20^. We thus sought to determine which source of intracellular ROS plays an essential role in the EA-mediated pro-apoptotic signaling using specific inhibitors for ROS generation from these sources, Nox inhibitor Apocynin (Apo) and mitochondria-specific antioxidant mito-TEMPO (MT). We found that as well as NAC, Apo, but not MT, strongly suppressed EA-mediated enhancement of CDDP-induced cell death (Fig. 4d), p38/JNK activation (Fig. 4e) and ROS generation (Fig. 4f,g). Moreover, EA-dependent upregulation of CDDP-induced ROS generation was not reversed by knocking out either *p53* or *ASK1* (see Supplementary Fig. S5 online), suggesting that upregulated ROS generation is an upstream event of p53- and ASK1-dependent pro-apoptotic signaling mediated by EA. These data collectively suggest that in response to CDDP, EA enhances the Nox-dependent ROS generation, and thereby promotes activation of the ASK1-p38/JNK MAPK pathway, and ultimately, p53-dependent cell death. In agreement with this notion, we found that ASK1 expression was not increased, but rather marginally downregulated by EA pretreatment, before or after CDDP treatment (see Supplementary Fig. S6 online), suggesting that EA increases ROS-dependent ASK1 activation, but not its expression, by CDDP treatment.

### RIP1 is required for the EA-mediated increase in ROS generation in response to CDDP

**Figure 5:**
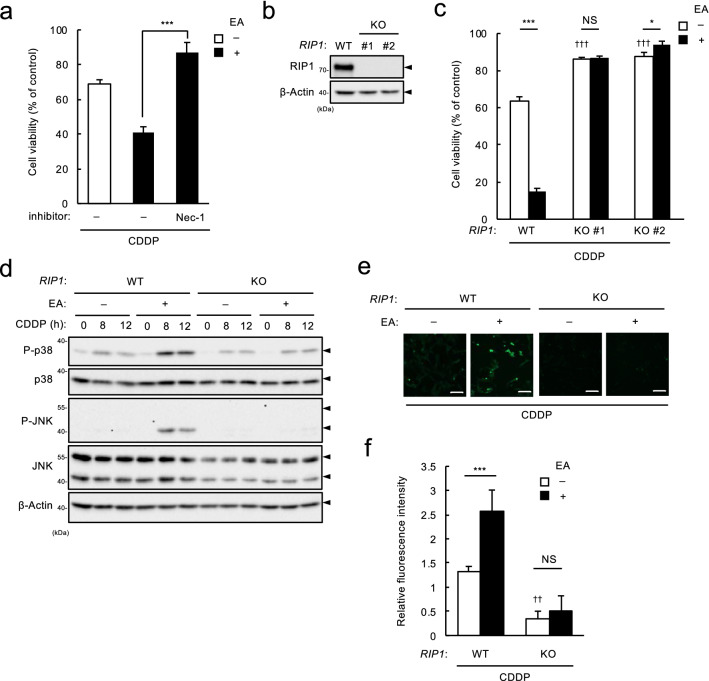
RIP1 plays a critical role in EA-mediated pro-apoptotic signaling in response to CDDP. (**a**) U2OS cells were pretreated with or without 200 µM EA for 11.5 h, treated with RIP1 inhibitor Necrostatin-1 (Nec-1, 30 μM) for 0.5 h, and then stimulated with 40 μM CDDP for 24 h, subjected to cell viability assay. Data shown are the mean ± SD (n = 3). (**b**) *RIP1* WT and KO U2OS cells were lysed and subjected to immunoblotting with the antibodies against RIP1 and β-actin. (**c**) *RIP1* WT and KO U2OS cells were pretreated with or without 200 μM EA for 12 h, and then treated with 40 μM CDDP for 24 h, subjected to cell viability assay. Data shown are the mean ± SD (n = 3). ^†††^*p* < 0.001 (vs. WT cells without EA pretreatment). (**e**) *RIP1* WT and KO U2OS cells were pretreated with or without 200 µM EA for 12 h, and then stimulated with 40 μM CDDP for 0, 8 and 12 h. Cell lysates were subjected to immunoblotting with the indicated antibodies. (**f** and **g**) *RIP1* WT and KO U2OS cells were pretreated with or without 200 µM EA for 12 h, and then stimulated with 40 μM CDDP for 6 h, followed by incorporation of a ROS-sensitive fluorescent probe DCFH-DA for 30 min. Green fluorescence was observed (**f**) and relative ROS levels were calculated (**g**), shown as mean ± SD (n = 3, normalized to the ROS level in the cells without fatty acid and CDDP). Scale bar, 100 µm. ^††^*p* < 0.01 (vs. WT cells without EA pretreatment).

To clarify a detailed mechanism for EA-mediated increase in CDDP-induced ROS generation via Nox, we focused on receptor-interacting protein 1 (RIP1), because a RIP1 specific inhibitor necrostatin-1 (Nec-1)^21^ strongly suppressed the EA-mediated enhancement of CDDP-induced cell death (Fig. 5a). RIP1 is a component of tumor necrosis factor receptor 1 (TNFR1) complex that is recruited to the complex in response to TNF-α stimulation, and regulates inflammatory responses and cell death^22^; meanwhile, notably, RIP1 is known to promote ROS generation by increasing formation and activation of the Nox1 complex^23^. Intriguingly, several lines of evidence have demonstrated that RIP1 participates in CDDP-induced ROS generation and cell death^24–26^. Therefore, we further examined the role of RIP1 in EA-mediated pro-apoptotic effect during CDDP-induced DNA damage by establishing *RIP1* KO U2OS cell lines (Fig. 5b). As expected, we observed that RIP1 deficiency dramatically suppressed EA-mediated promotion of cell death by treatment with CDDP (Fig. 5c), but not that with Dox (see Supplementary Fig. S7 online). Furthermore, *RIP1* knockout also canceled EA-dependent increase in p38/JNK activation (Fig. 5d) and ROS generation (Fig. 5e,f) induced by CDDP. Notably, even in the absence of EA, CDDP-induced ROS-generation and cell death were significantly prevented (Fig. 5c,f). Altogether, these data suggest that in consistent with previous reports, RIP1 contributes to CDDP-induced ROS generation and cell death^24–26^, and more importantly, that RIP1 plays a key role for pro-apoptotic effect of EA during CDDP-induced DNA damage through enhancing Nox-dependent ROS generation.

## Discussion

**Figure 6:**
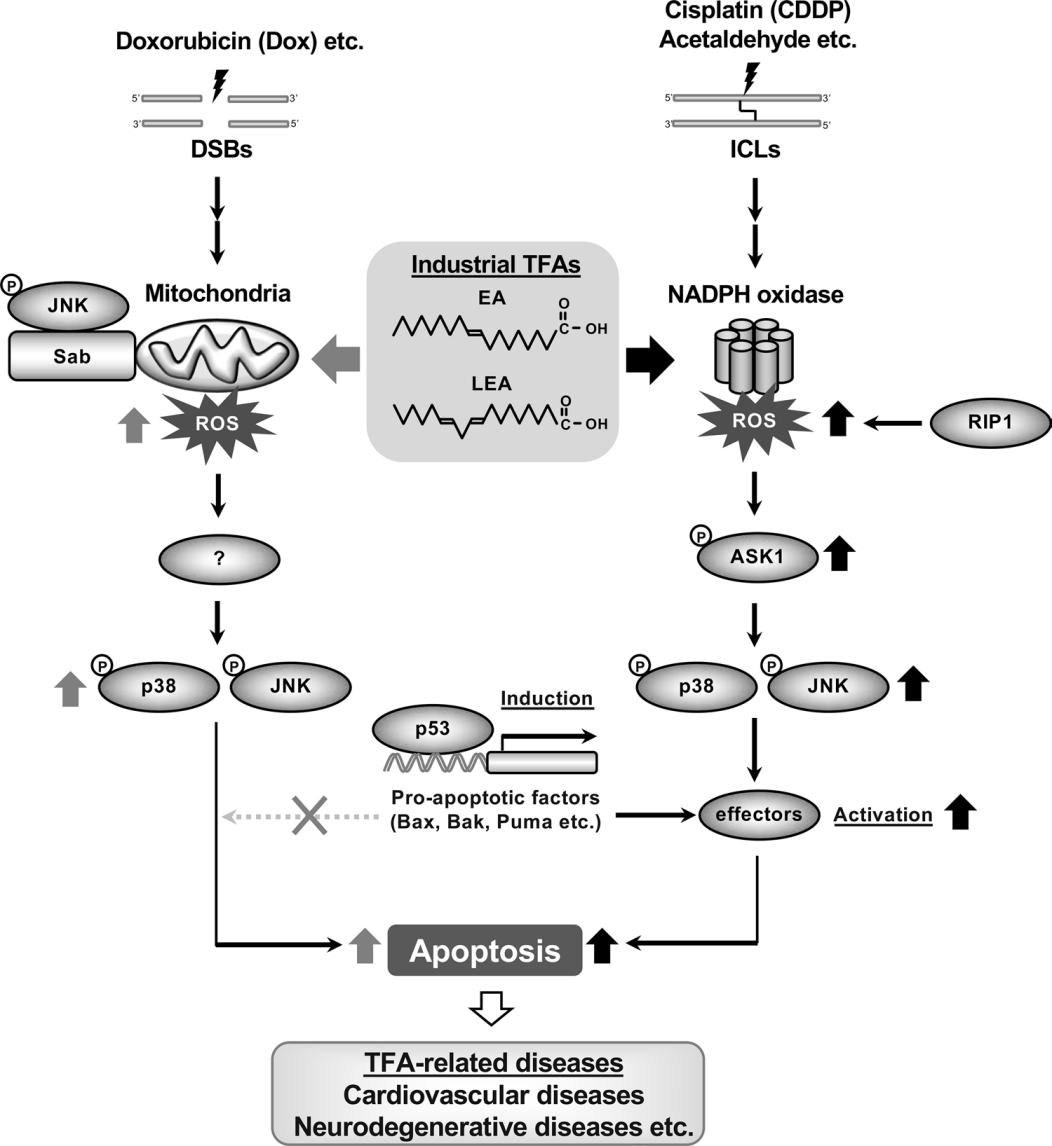
A schematic model for two distinct EA-mediated pro-apoptotic signaling pathways in response to DNA damage. Industrial TFAs such as EA and LEA promote cell death triggered by Dox (a DSB-inducing agent) and CDDP (an ICL-inducing agent) through two distinct pro-apoptotic signaling pathways. Upon Dox exposure, TFAs enforce mitochondrial ROS generation via the mitochondrial JNK-Sab (a JNK adaptor protein localized at outer membrane of mitochondria) axis, which in turn enhance JNK/p38 MAPK activation, and consequently promote apoptosis independently of ASK1 and p53^12^. On the other hand, upon CDDP exposure, TFAs drive Nox/RIP1-dependent ROS generation, and thereby enhance activation of the ASK1-p38/JNK MAPK pathway, leading to increased p53-dependent apoptosis. The latter mechanism possibly accounts for TFA-mediated promoting effect on cell death induced by acetaldehyde, a major endogenous source of ICLs that accumulates by unhealthy lifestyle behaviors, such as cigarette smoking and alcohol drinking, or decreased acetaldehyde metabolism. Through these mechanisms, TFAs may contribute to the development and progression of TFA-related diseases, including cardiovascular diseases and neurodegenerative disorders, closely related to DNA damage along with individual difference in lifestyle factors and acetaldehyde metabolism.

Our data reveal that TFAs such as EA, but not their corresponding *cis* isomers, promote CDDP-induced apoptosis in a p53-dependent manner by potentiating Nox/RIP1-dependent ROS generation and subsequent activation of the ASK1-p38/JNK MAP kinase pathway, which is distinct from the p53-independent pro-apoptotic signaling that was observed in Dox-induced apoptosis in our previous report^12^. Despite the difference in the pro-apoptotic mechanism, industrial TFAs such as EA and LEA, but not TVA (the most abundant ruminant TFA), promoted apoptosis induced by CDDP as well as that induced by Dox^12^. These results collectively suggest that industrial TFAs particularly act as an apoptosis promoter by two distinct mechanisms in response to Dox-induced DSBs and CDDP-induced ICLs, which may account for their specific epidemiological association with TFA-related diseases including CVDs^27,28^ and NDs^4,29,30^ (Fig. 6).

According to the types of DNA lesions, cells utilize different DNA damage sensing systems and DDRs. For instance, a serine/threonine protein kinase, ataxia-telangiectasia mutated (ATM), is primarily activated by DSBs, while another protein kinase ATR is activated in response to DNA replication forks stalled by ICLs, which defines the difference of downstream signaling pathways between cells treated with Dox and CDDP, DSB- and ICL-inducing agents, respectively^31^. Indeed, we obtained data suggestive of such a difference between the two cases of DNA-damaging agents; in Dox-treated cells, γH2AX was accumulated more abundantly than in CDDP-treated cells, and *ATM* knockdown enhanced pro-apoptotic action of EA presumably due to increase in DSB repair failure, but not in CDDP-treated cells (see Supplementary Fig. S8 online). In this study, we found a clear distinction in RIP1 dependency of cell death induced by these two agents; in RIP1-deficient U2OS cell, CDDP-induced cell death was largely suppressed with or without EA (Fig. 5c), whereas Dox-induced cell death was observed at the same level as in WT cells (see Supplementary Fig. S7 online). These data suggest a specific requirement of RIP1 for cell death induced by CDDP, in agreement with the previous reports^25,32,33^. Moreover, we showed that RIP1 deficiency strongly inhibited CDDP-induced ROS generation irrespective of the presence of EA (Fig. 5e,f), implicating a molecular link of RIP1 to Nox-dependent ROS generation initiated by CDDP-induced ICLs. Notably, CDDP is widely used as a chemotherapeutic drug for many types of cancers, but possesses numerous adverse effects on health, including ototoxicity and nephrotoxicity due to hyperactivation of Nox^34,35^. CDDP-induced Nox hyperactivation has been observed not only in cochlear cells and kidney cells, but also in various types of cancer cells, which has been attributed to Nox subunit upregulation^36,37^. Since NF-κB involves transcriptional regulation of multiple Nox subunits^38^ and RIP1 contributes to DNA damage-induced NF-κB activation as a component of the complex so-called PIDDosome^39^, RIP1 might promote upregulation of Nox subunits in response to CDDP. Intriguingly, a previous report demonstrated that CDDP enhances Nox-dependent ROS generation in prostate cancer cell lines without affecting Nox subunit expressions^40^, implying the existence of a more direct mechanism of CDDP-induced Nox activation. Therefore, RIP1 might participate in Nox-dependent ROS generation in response to CDDP by forming complex with Nox subunits, as well as that in response to TNF-α^23^. More importantly, we demonstrated a critical role of RIP1 in EA-mediated enhancement of Nox-dependent ROS generation, p38/JNK MAPK activation and ultimately apoptosis (Fig. 5). TFAs may augment CDDP-induced Nox activation by increasing its subunit expressions or enzymatic activities via a mechanism involving RIP1, which should be clarified in the future studies.

Besides RIP1, we also identified p53 and ASK1 as key molecules that define the two distinct pro-apoptotic signaling pathways mediated by EA; both p53 and ASK1 are necessary for EA-mediated pro-apoptotic effect on CDDP-induced cell death, but not for Dox-induced cell death (Figs. 2, 3)^12^. Mechanistically, EA enhanced CDDP-induced activation of p38/JNK MAP kinases that requires Nox-dependent ROS generation and ASK1, and ultimately cell death, possibly through increasing the p53-dependent mitochondrial apoptosis (Figs. 2, 3). Although p53 is a major substrate of p38/JNK MAP kinases, we did not find any significant positive effect of EA on the phosphorylation of p53 at Ser15 and Ser20 (Fig. 3f), p53 translocation to the nucleus and mitochondria (Fig. 2d,f), nor transcriptional levels of p53 target genes (Fig. 2e), which might be due to the difference of their activation kinetics; upon CDDP treatment, p53 was induced and translocated to the nucleus from 4 h (Fig. 3f), while EA-mediated p38/JNK hyperactivation was observed from 8 h (Fig. 3b). Thus, it is likely that in response to CDDP, p53 is firstly activated and induces expression of pro-apoptotic factors that are substrates of p38/JNK, such as Bax, Bcl-2-associated agonist of cell death (Bad), and Bcl-2-like protein 11 (Bim)^41^, which are then hyperphosphorylated and consequently promote apoptosis in the presence of EA.

Cells are exposed to various ICL-inducing agents, such as byproducts of endogenous metabolic processes (e.g. acetaldehyde and malondialdehyde) and exogenous mutagens (e.g. chemotherapeutic agents)^42,43^. In this study, we mainly used CDDP as a typical ICL-inducing chemotherapeutic agent. Meanwhile, the most common natural source of ICLs is acetaldehyde, a catabolite of ethanol. Acetaldehyde can be generated endogenously in a variety of metabolic processes, and forms multiple types of adducts with DNA and proteins including ICLs, leading to genomic instability and carcinogenesis^44^. In order to avoid the toxicity, endogenously produced acetaldehyde is immediately catabolized by aldehyde dehydrogenase 2 (ALDH2), or once it forms ICLs, they are repaired specifically by Fanconi anemia complementation group (FANC) proteins that constitute the Fanconi pathway^43,45^. Indeed, hematopoietic cells that lack FANC proteins are hypersensitive to acetaldehyde treatment, resulting in increased cell death; mice deficient in both *FANCD2* and *ALDH2* show various developmental defects including embryonic lethality and bone marrow failure owing to genotoxicity caused by aldehyde accumulation^46,47^. Thus, endogenously produced acetaldehyde is harmful due to, at least partly, formation of ICLs, but promptly eliminated by the detoxification system involving ALDH2 and FANC proteins. However, unhealthy lifestyle factors, such as chronic alcohol consumption and cigarette smoking, are known to cause accumulation of acetaldehyde beyond the capacity of the detoxification system, and increase the risk of various diseases such as metabolic diseases, NDs, and CVDs^48–50^. Interestingly, all of the above diseases are associated with TFA consumption as well, and we therefore speculate that TFA intake might raise the risk of these TFA-related diseases in a synergetic manner with acetaldehyde accumulation by alcohol consumption or cigarette smoking, or with abnormalities in detoxification system caused by deficiency of ALDH2 and FANC proteins. Since, to the best of our knowledge, no experimental or epidemiological study has examined this assumption, we performed analyses to examine whether EA has a pro-apoptotic role in response to acetaldehyde. We indeed found that EA pretreatment strongly accelerated acetaldehyde-induced cell death in U2OS cells, which was partially but significantly reversed by p53 deficiency (see Supplementary Fig. S9 online). These data implicate that EA promotes p53-dependent cell death in response to acetaldehyde-induced ICLs, likely via the pro-apoptotic mechanism involving Nox/RIP1-dependent p38/JNK hyperactivation, which was observed in CDDP-induced cell death (Fig. 6). Notably, whereas acetaldehyde induces variety types of adducts with DNA and proteins other than ICLs, CDDP can specifically induce ICLs^51^. In this study, taking advantage of such property of CDDP, we thus provide a pro-apoptotic mechanism of TFAs triggered by ICLs. Our study may contribute to further understanding of the underlying pathological mechanisms of TFA-related diseases that are closely linked to DNA damage, especially ICLs, in conjunction with individual difference in lifestyle factors and acetaldehyde metabolism.

## Methods

### Reagents

Doxorubicin (Dox) was purchased from Sigma. Cisplatin (CDDP), acetaldehyde, SP600125 (SP) and N-acetylcysteine (NAC) were purchased from Wako. SB203580 (SB), Apocynin (Apo), mito-TEMPO (MT) and Necrostatin-1 (Nec-1) were purchased from Santa Cruz. z-VAD-fmk was purchased from Peptide Institute.

### Cell culture

U2OS, HEK293T and TIG-3 cells, and RAW264.7 cells were cultured in Dulbecco's Modified Eagle's medium (Nakalai) and RPMI 1640 medium (Nakalai), respectively, containing 10% heat-inactivated fetal bovine serum and 1% penicillin–streptomycin solution in 5% CO2 at 37 °C.

### siRNA knockdown

siRNA targeting human *ATM* was obtained from GeneDesign. U2OS cells were transfected with 10 nM non-targeting siRNA pool (Dharmacon) as control or *ATM* siRNA using Lipofectamine RNAiMAX Transfection Reagent (Invitrogen), according to the manufacturer’s instructions. *ATM* siRNA sequence was 5′-AAGCGCCUGAUUCGAGAUCCU-3’.

### Preparation and treatment of fatty acids

Fatty acids including PA, OA (Nacalai tesque), EA (Sigma), LA, LEA (Cayman Chemical), CVA and TVA (Olbracht Serdary Research Laboratories) were prepared as described previously^52^. Briefly, fatty acids were dissolved in 0.1 N NaOH at 70 °C, and then conjugated with fatty acid-free BSA (Wako, pH 7.4) at 55 °C for 10 min to make 5 mM BSA-conjugated fatty acid stock solutions containing 10% BSA. Cells were treated with various concentrations of BSA-conjugated fatty acids by diluting stock solutions in medium without fetal bovine serum (final BSA concentration was set to 1%).

### Immunoblot analysis

Cells were lysed in ice-cold lysis buffer containing 20 mM Tris–HCl, pH 7.4, 150 mM NaCl, 1% Triton-X100, 10% Glycerol, and 1% protease inhibitor cocktail (Nacalai tesque). After centrifugation, the cell extracts were resolved by SDS-PAGE, and were analyzed as described previously^53^. In Figs. 2d and 3f, nuclear extraction was performed as described previously^54^, whereas in Fig. 2f, mitochondria were isolated as described previously^12^, and subjected to immunoblot analysis. The antibodies used for immunoblotting were against phospho-p38, p38, phospho-JNK, JNK, caspase-3, caspase-9, p53, phospho-p53 (Ser15 and Ser20) (Cell Signaling), β-actin (Wako), Cox4 (Proteintech), ASK1, ATM, γH2AX, H2AX and lamin A/C (Santa cruz). The blots were developed with ECL (Merck Millipore), and detected with ChemiDoc Touch Imaging System (BioRad).

### Immunocytochemistry

Immunocytochemistry was performed as described previously^55^, using antibodies against γH2AX (Santa cruz) and β-actin (Proteintech), and Fluoro-KEEPER Antifade Reagent (Non-Hardening Type with DAPI, Nakalai) for mounting medium. The immunostained samples were observed with either Zeiss LSM800 or Olympus Fluoview FV1000 confocal fluorescence microscope.

### Cell viability assay

U2OS cells were seeded on 96-well plates. After any stimulation or treatment, cell viability was determined using Cell Titer 96 Cell Proliferation Assay (Promega), according to the manufacturer's protocol. The absorbance was read at 492 nm using a microplate reader (iMark, Bio-Rad). Data are normalized to control without stimulus, unless noted otherwise.

### qRT-PCR analysis

Total RNA was extracted using Sepasol RNA I Super (Nacalai Tesque) and then was reverse transcripted into cDNA with High-Capacity cDNA Reverse Transcription Kit (Applied Biosystems). mRNA levels were measured by qRT-PCR using Luna Universal qPCR Master Mix (New England Biolabs), and normalized with those of gapdh. Sequences of primers used in this are: p21-forward, 5′-GAGGCCGGGATGAGTTGGGAGGAG-3′ and p21-reverse, 5′-CAGCCGGCGTTTGGAGTGGTAGAA-3′; Puma-forward, 5′-TTGTGCTGGTGCCCGTTCCA-3′ and Puma-reverse, 5′-AGGCTAGTGGTCACGTTTGGCT-3′; gapdh-forward, 5′-AACAGCCTCAAGATCATCAGC-3′, and gapdh-reverse, 5′-GGATGATGTTCTGGAGAGCC-3’.

### Bioimaging and quantification of ROS

Bioimaging and quantification of ROS was performed as described previously^12^. U2OS cells were seeded on glass plates. After stimulation, cells were treated with 10 μM DCFH-DA (Sigma) for 30 min at 37 °C. Intracellular ROS generation was observed using a Zeiss LSM800 laser confocal microscope (Carl Zeiss) and the images were processed with Zen software. Data are shown as men ± SD of relative fluorescence intensity from three different fields of view, which was calculated by dividing total green fluorescence (background was subtracted) by cell numbers using Image J.

### Generation of knockout cell lines

*p53-, ASK1*-, and *RIP1-*knockout cells were generated using the CRISPR/Cas9 system ^56,57^. guide RNAs (gRNAs) were designed to target exon 5 of *p53* gene (5′-ACCATGAGCGCTGCTCAGAT-3′), exon 3 of *ASK1* gene (5′-GGGGCAGGATACGGACTGCA-3′) and exon 3 of *RIP1* gene (5′-CTTCCTCTATGATGACGCCC-3′) using CRISPRdirect^58^. gRNA-encoding oligonucleotides were cloned into lentiCRISPRv2 plasmid^59^, and the plasmids were transfected with HEK293T cells together with a packaging plasmid psPAX2 and an envelope plasmid pVSV-G. The virus-containing supernatants were collected and used for infecting U2OS cells, and then infected cells were selected with puromycin and cloned by limiting dilution. To determine the mutations of *p53* and *ASK1* in cloned cells, genomic sequences around the target regions were analyzed by PCR-direct sequencing using the following primers: 5′-TCCAAATACTCCACACGCAA-3′ and 5′-CTACAAGCAGTCACAGCATATG-3′ for *p53*; 5′-ACACTCACCCTTTCGGCATT-3′ and 5′-GGGGTGCTGCTTCTTTCTCT-3′ for *ASK1*; 5′-TCTACCTCGGCTTTCAGCAC-3′ and 5′-TCTAACGCTTCTGGCCTGTG-3′ for *RIP1*.

### Nuclear extraction

Nuclear extraction was performed as described previously^54^. Cells were lysed in ice-cold lysis buffer containing 10 mM HEPES (pH 7.5), 10 mM KCl, 0.1 mM EGTA, 0.1 mM EDTA, 1 mM DTT, and 1% protease inhibitor cocktails (Nacalai Tesque) for 15 min. Cell lysates were added 1% NP-40, and then centrifuged at 4 °C at 2,500 rpm for 3 min. After the supernatants containing cytoplasmic fraction were removed, the pellets were suspended in ice-cold lysis buffer containing 20 mM HEPES (pH 7.5), 400 mM NaCl, 1 mM EGTA, 1 mM DTT, and 1% protease inhibitor cocktails for 15 min with vortexed every 5 min. Cell lysates were then centrifuged at 4 °C at 15,000 rpm for 15 min, and then the supernatants were collected as nuclear fractions.

### Statistical analysis

All the values are expressed as the mean ± standard deviation (S.D.), and statistical analysis was performed using GraphPad Prism software. All experiments were repeated at least three independent times. Two groups were compared using two-tailed Student’s t-test. Multiple-group comparisons were conducted using the one-way ANOVA analysis followed by Tukey–Kramer test. Significant differences were represented as follows: NS, not significant; *p < 0.05; **p < 0.01; ***p < 0.001; ^††^*p* < 0.01; ^†††^*p* < 0.001.

## Supplementary Information

Supplementary Figures
