# Supplementary material for: trans-Fatty acids promote p53-dependent apoptosis triggered by cisplatin-induced DNA interstrand crosslinks via the Nox-RIP1-ASK1-MAPK pathway
Source: Sci Rep. 2021 May 14;11:10350. doi: 10.1038/s41598-021-89506-8 (PMC8121903; doi:10.1038/s41598-021-89506-8)
Supplement: Supplementary file 1 — Supplementary Figures [file 41598_2021_89506_MOESM1_ESM.pdf]

Supplementary Information

trans-Fatty acids promote p53-dependent apoptosis triggered by cisplatin-induced DNA interstrand crosslinks via the Nox-RIP1-ASK1-MAPK pathway

Yusuke Hirata, Miki Takahashi, Yuto Yamada, Ryosuke Matsui, Aya Inoue, Ryo Ashida, Takuya Noguchi, and Atsushi Matsuzawa

Included materials

- **Figure S1.**
- **Figure S2.**
- **Figure S3.**
- **Figure S4.**
- **Figure S5.**
- **Figure S6.**
- **Figure S7.**
- **Figure S8.**
- **Figure S9.**
- **Figure S10.**

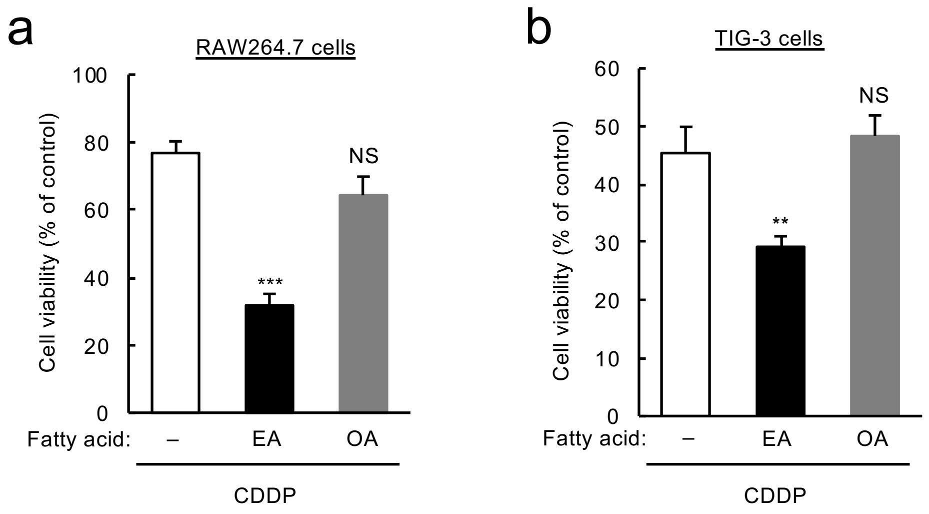

Figure S1. EA promotes CDDP-induced cell death in various cell lines
(a and b) RAW264.7 cells (a) and TIG-3 cells (b) were pretreated with or without 200 μM OA or EA for 12 h, and then stimulated with CDDP at 40 μM (a) or 80 μM (b) for 24 h, subjected to cell viability assay. NS, not significant; **p < 0.01; ***p < 0.001 (vs control cells without fatty acid).

Hirata et al., Fig. S1.

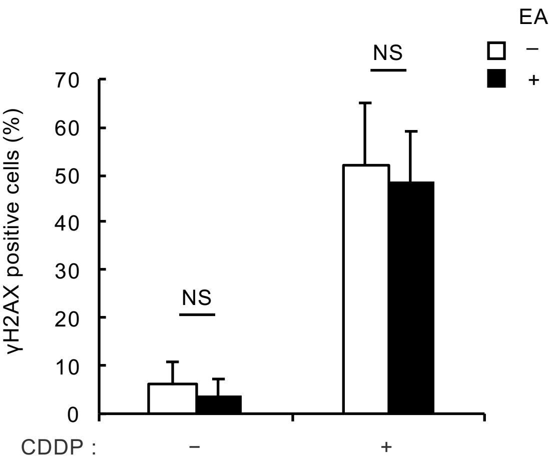

Figure S2. EA does not affect γH2AX accumulation induced by CDDP
Quantitative analysis for the immunofluorescence data in Fig. 2a. Cells with 5 or more γH2AX foci were defined as γH2AX positive cells since most of the CDDP-untreated cells exhibited 2–4 foci per cell. The graph shows the percentage of γH2AX positive cells (mean ± SD of 5 fields per group). NS, not significant.

Hirata et al., Fig. S2.

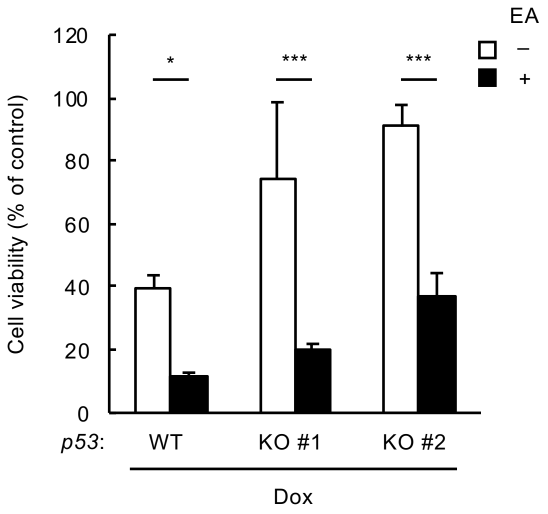

Figure S3. EA-mediated pro-apoptotic effect on Dox-induced cell death was not suppressed in *p53* KO cells

p53 WT and KO U2OS cells were pretreated with or without 200 μM EA for 12 h, and then treated with 0.5 μg/ml Dox for 24 h, subjected to cell viability assay. Data shown are the mean ± SD (n = 3). *p < 0.05; ***p < 0.001.

Hirata et al., Fig. S3.

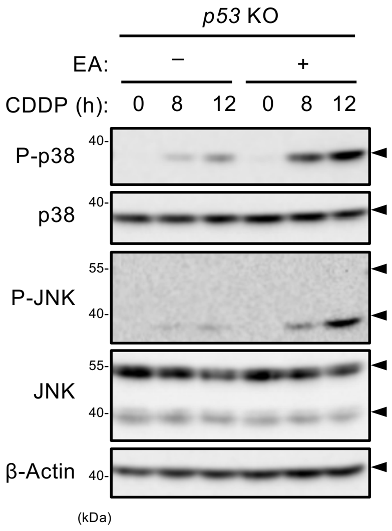

Figure S4. *p53* knockout does not affect EA-dependent hyperactivation of p38/JNK in response to CDDP

p53 WT and KO U2OS cells were pretreated with or without 200 μM EA for 12 h, and then stimulated with 40 μM CDDP for 0, 8 and 12 h. Cell lysates were subjected to immunoblotting with the indicated antibodies.

Hirata et al., Fig. S4.

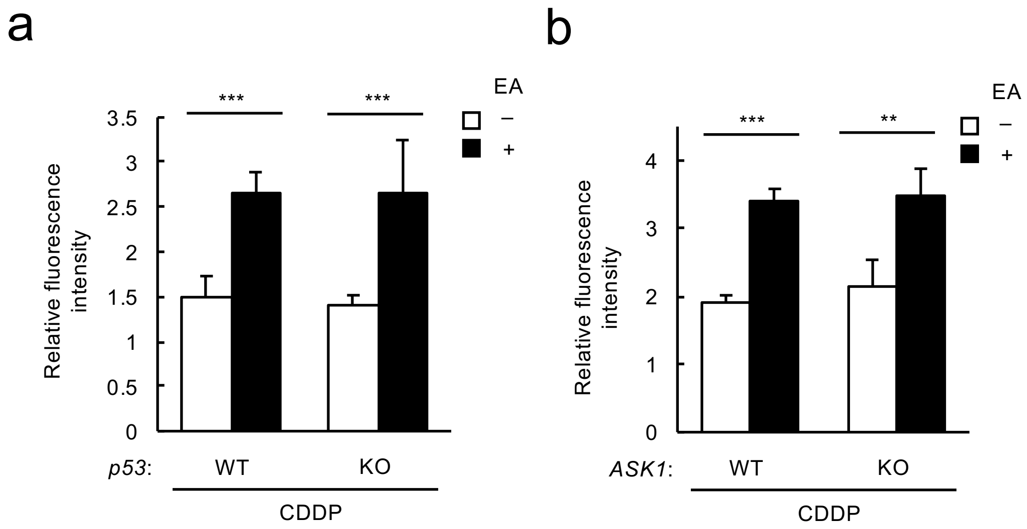

Figure S5. EA-mediated increase in CDDP-induced ROS generation was not affected in *p53* and *ASK1* KO cells

(a and b) WT, *p53* KO (a), and *ASK1* KO (b) U2OS cells were pretreated with or without 200 µM EA for 12 h, and then stimulated with 40 µM CDDP for 6 h, followed by incorporation of a ROS-sensitive fluorescent probe DCFH-DA for 30 min. Green fluorescence was observed and relative ROS levels were calculated, shown as mean ± SD (n = 3, normalized to the ROS level in the cells without fatty acid and CDDP). **p < 0.01; ***p < 0.001.

Hirata et al., Fig. S5.

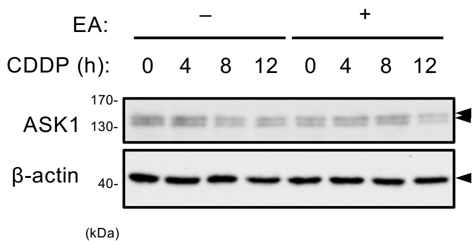

Figure S6. EA does not increase ASK1 expression in response to CDDP
U2OS cells were pretreated with or without 200 μM EA for 12 h, and then stimulated with 40 μM CDDP for 0, 4, 8 and 12 h. Cell lysates were subjected to immunoblotting with the indicated antibodies.

Hirata et al., Fig. S6.

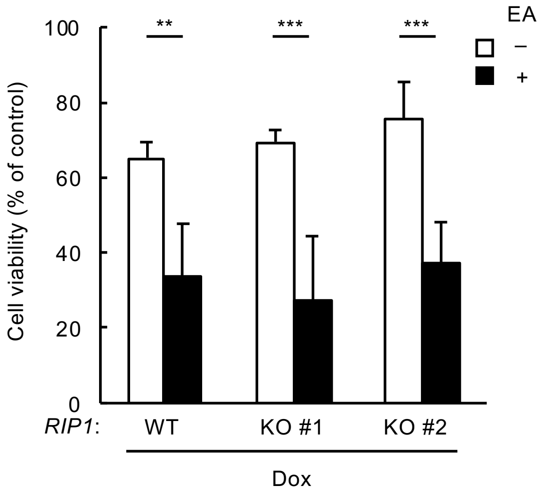

Figure S7. EA-mediated pro-apoptotic effect on Dox-induced cell death was not reversed in *RIP1* KO cells

RIP1 WT and KO U2OS cells were pretreated with or without 200 μM EA for 12 h, and then treated with 0.5 μg/ml Dox for 24 h, subjected to cell viability assay. Data shown are the mean ± SD (n = 3).

Hirata et al., Fig. S7.

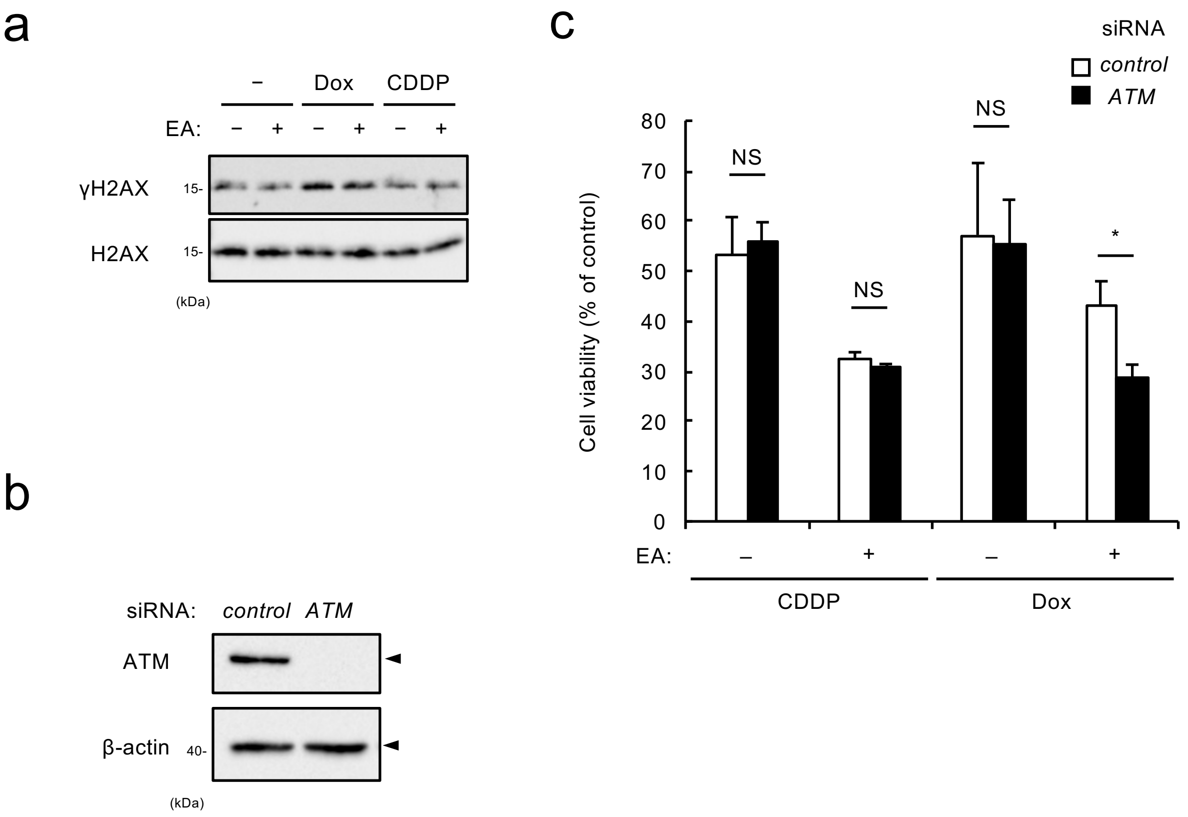

Figure S8. EA-mediated pro-apoptotic action in Dox-induced cell death is counteracted by the DSB-responsive kinase ATM

(a) U2OS cells were pretreated with or without EA for 12 h, and then stimulated with 0.5 μg/ml Dox or 40 μM CDDP for 0 and 6 h, subjected to immunoblotting with the indicated antibodies.

(b) U2OS cells transfected with siRNAs targeting non-targeting *control* or *ATM* for 48 h were lysed, and subjected to immunoblot analysis with the indicated antibodies for assessing knockdown efficiency of ATM.

(c) U2OS cells were transfected with siRNAs targeting non-targeting *control* or *ATM* for 48 h, pretreated with or without EA for 12 h, and then stimulated with 0.5 μg/ml Dox or 40 μM CDDP for 24 h, subjected to cell viability assay. NS, not significant; *$p < 0.05$.

Hirata et al., Fig. S8.

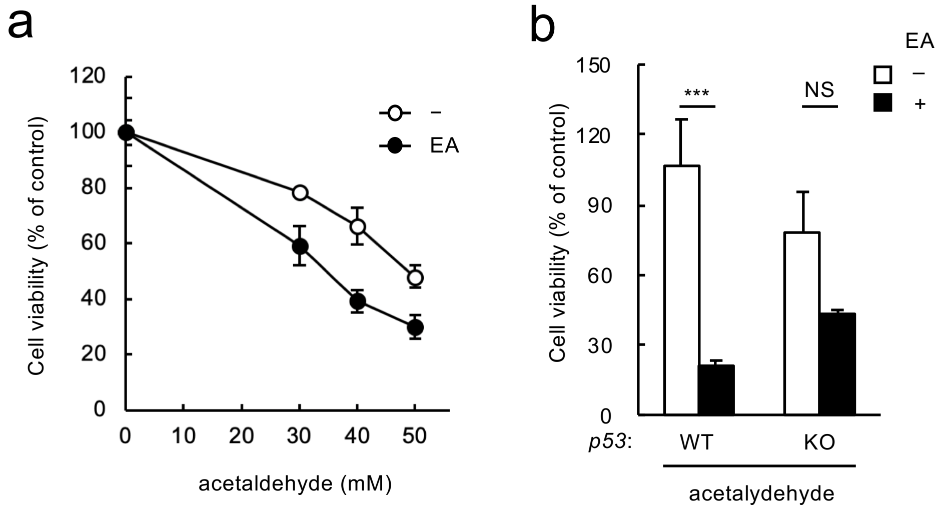

Figure S9. EA promotes acetaldehyde-induced cell death in a manner partially dependent on p53
(a) U2OS cells were pretreated with or without EA for 12 h, and then stimulated with various concentrations of acetaldehyde for 24 h, subjected to cell viability assay.
(b) *p53* WT and KO U2OS cells were pretreated with or without EA for 12 h, and then stimulated with 50 mM acetaldehyde for 24 h, subjected to cell viability assay. NS, not significant; ***p < 0.001.

Hirata et al., Fig. S9.

a

Fig. 1c

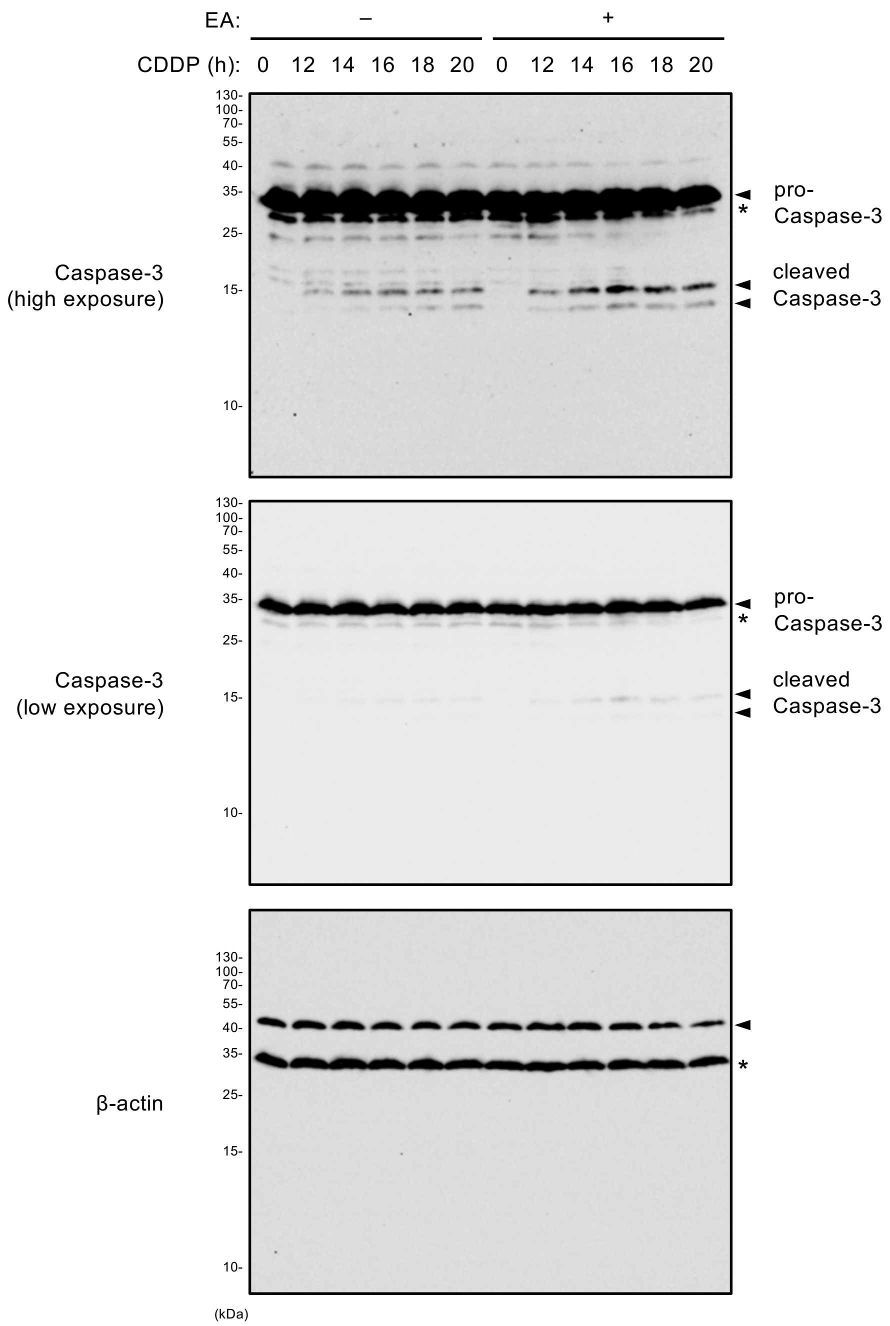

b

Fig. 2b

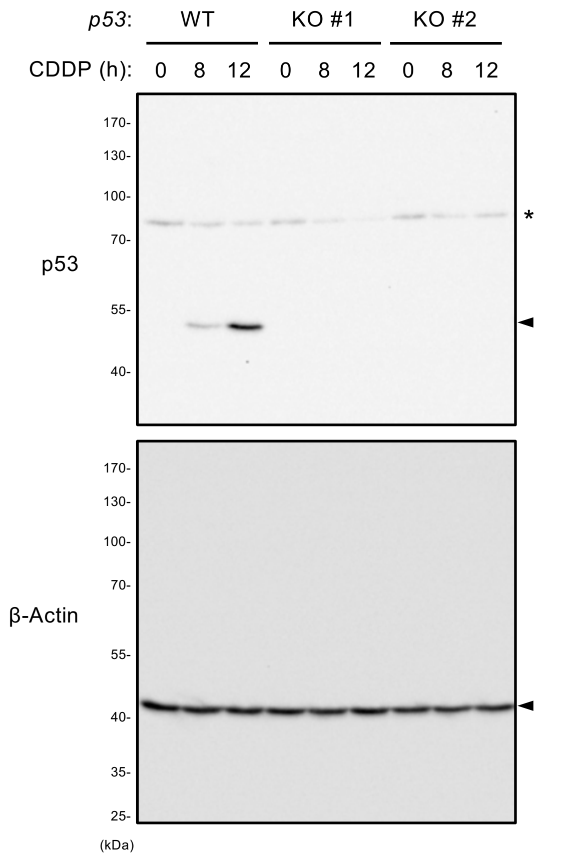

Hirata et al., Fig. S10.

c Fig. 2d

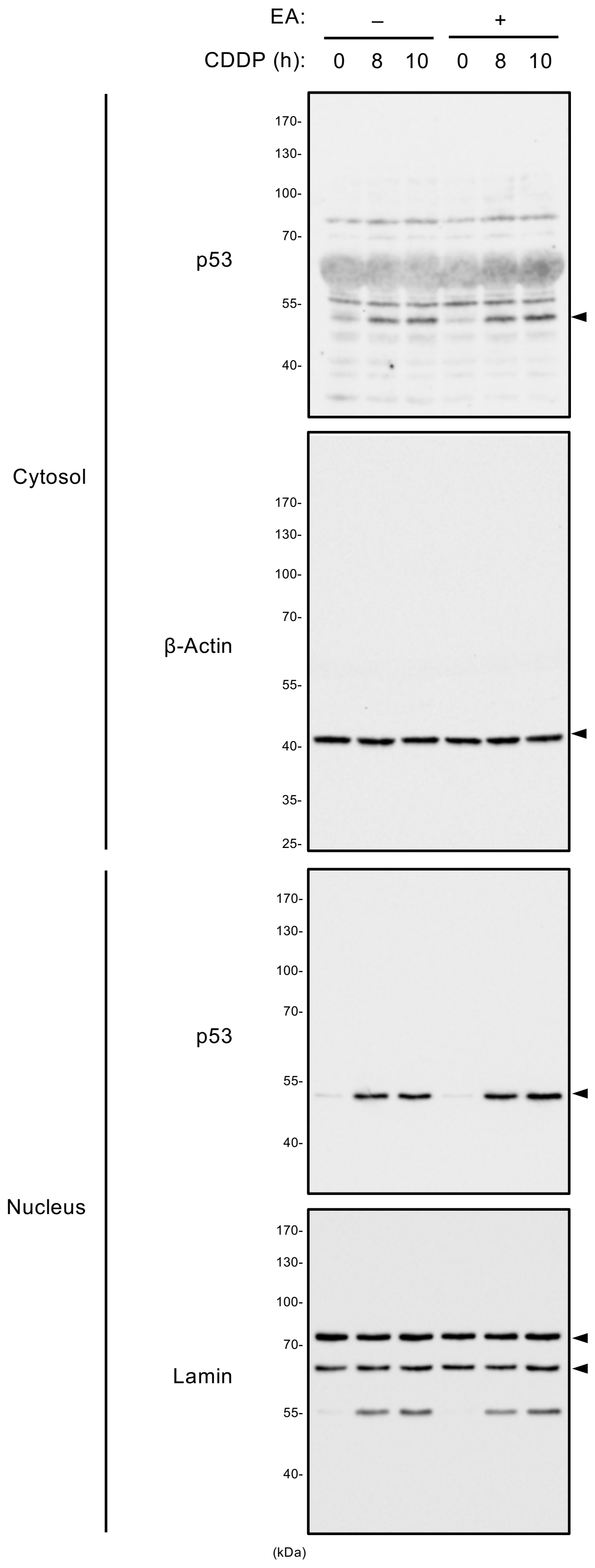

d Fig. 2f

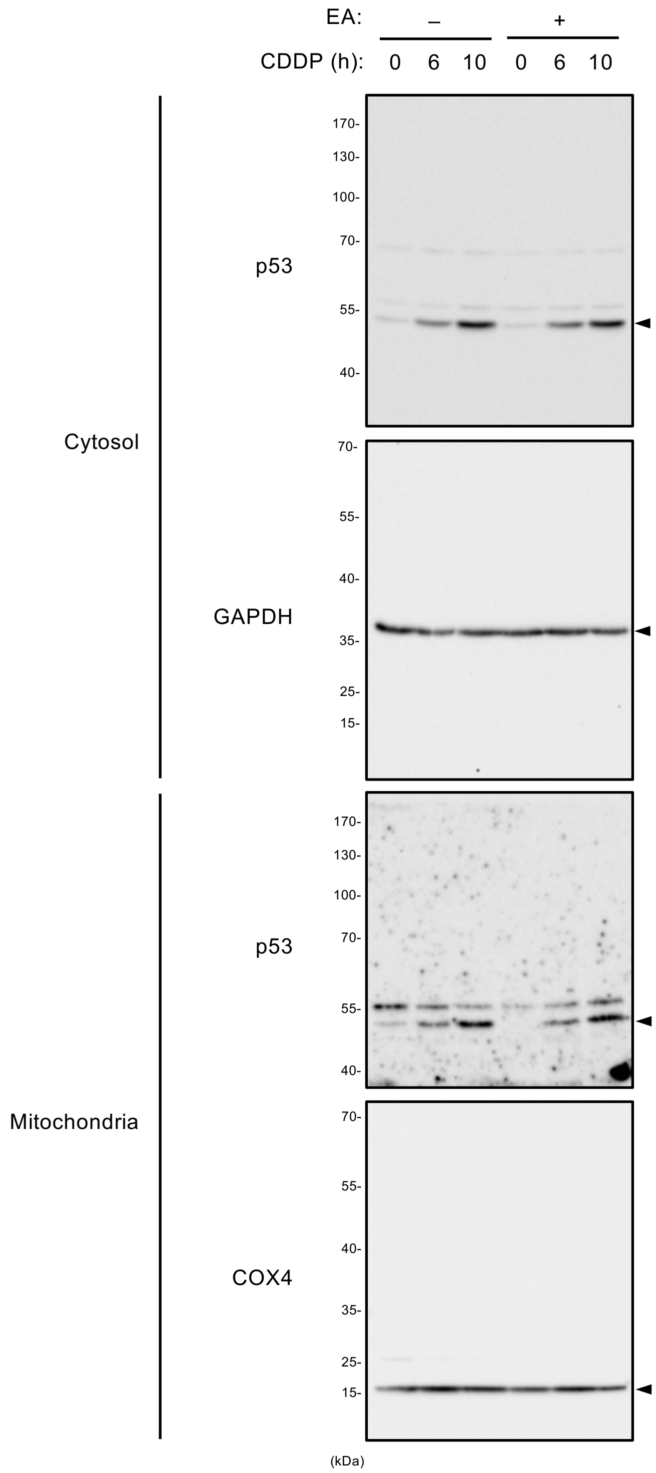

Hirata et al., Fig. S10.

e

Fig. 3b

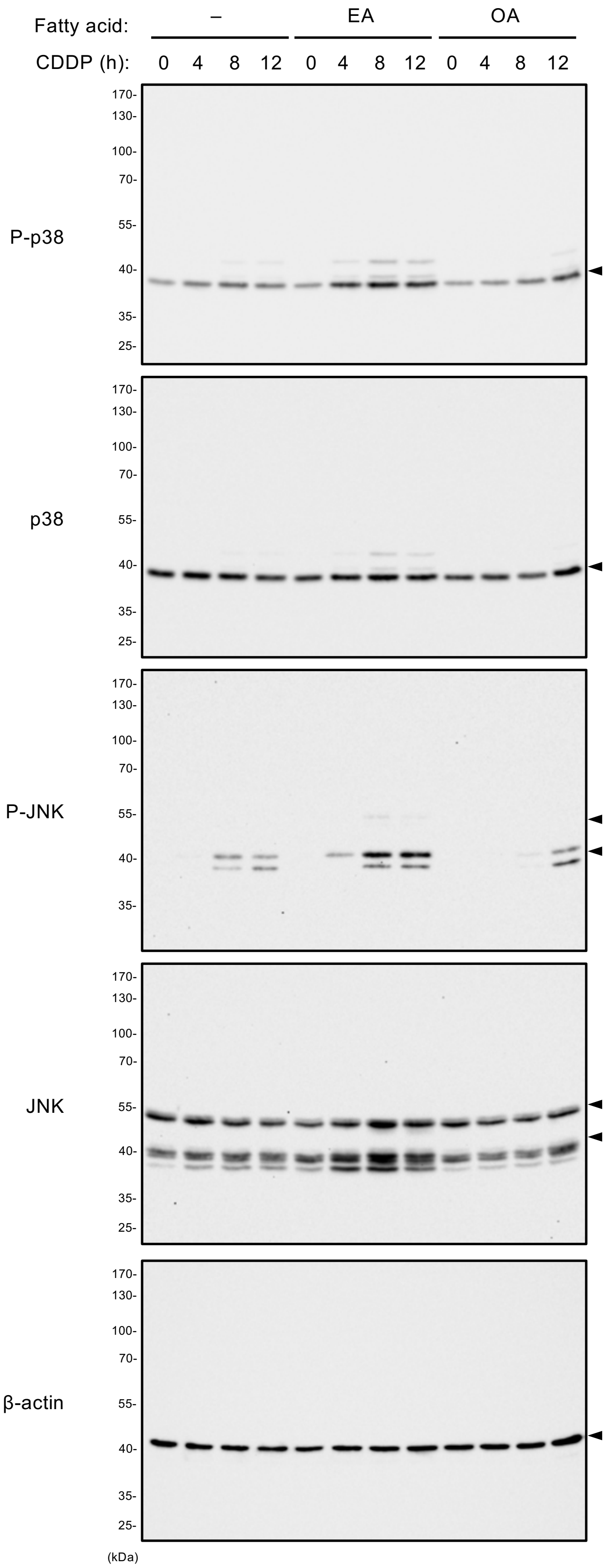

f

Fig. 3c

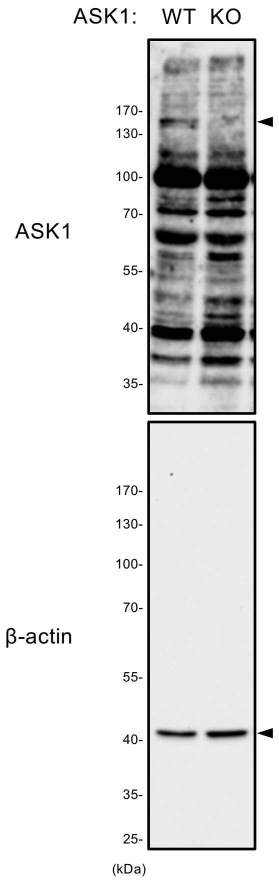

Hirata et al., Fig. S10.

g Fig. 3e

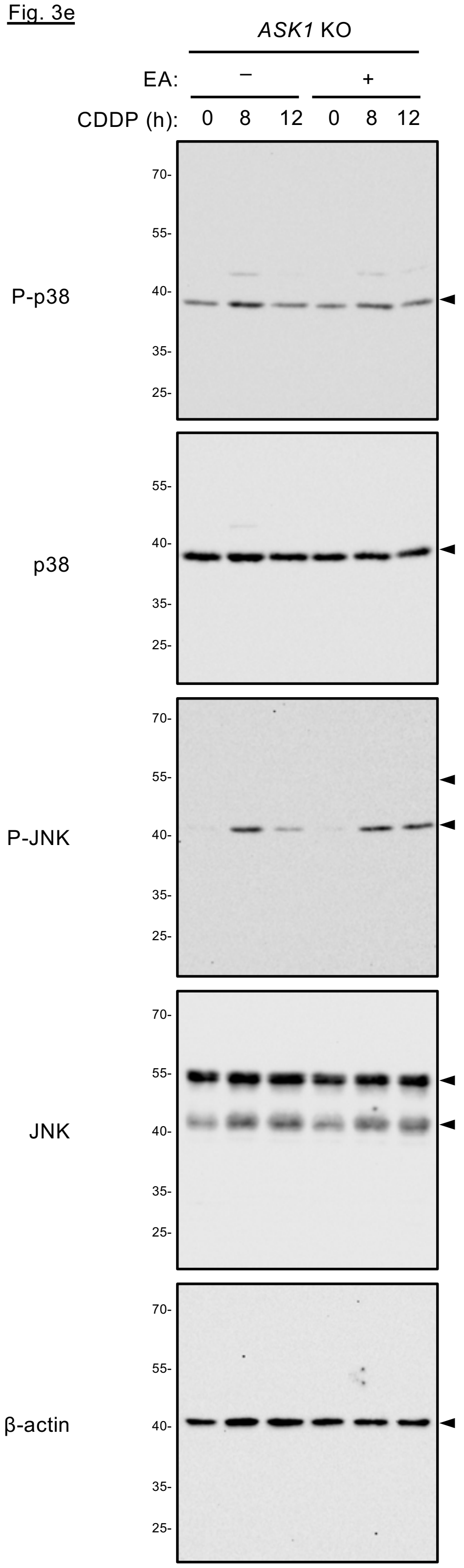

h Fig. 3f

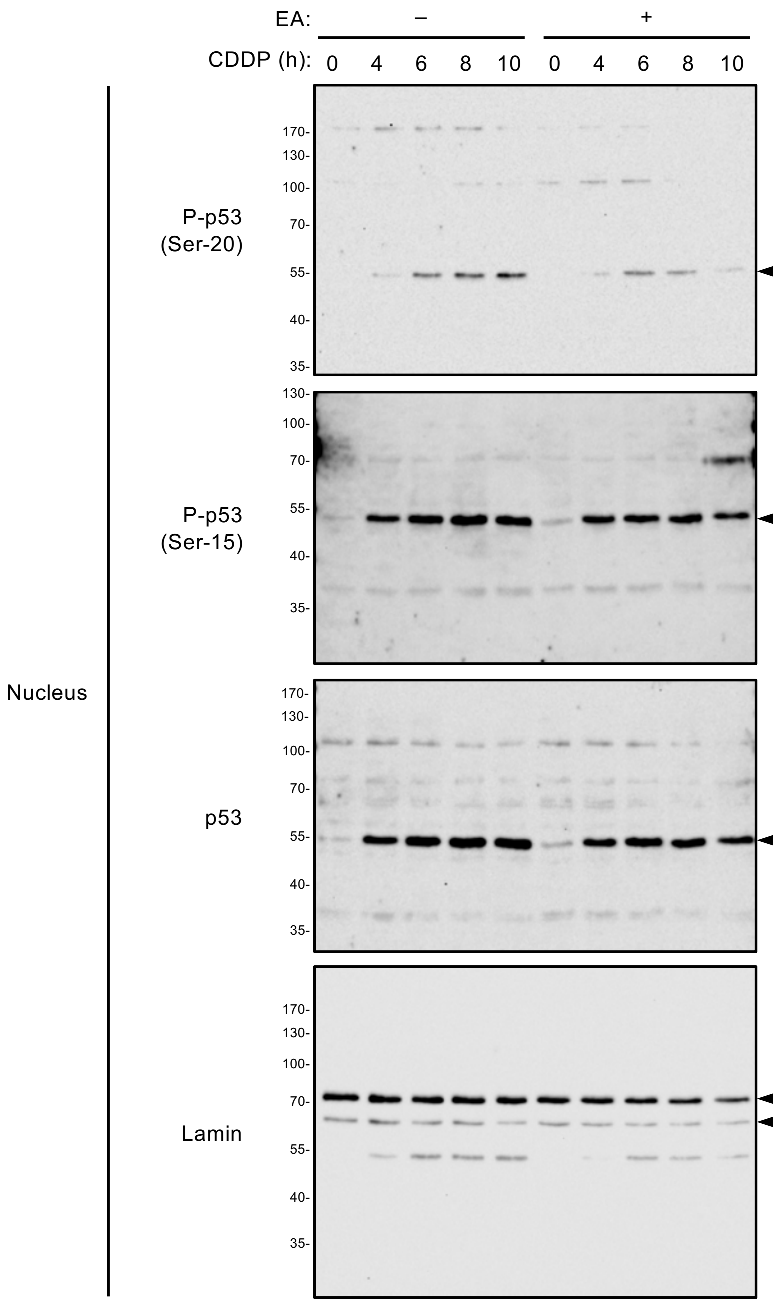

Hirata et al., Fig. S10.

i

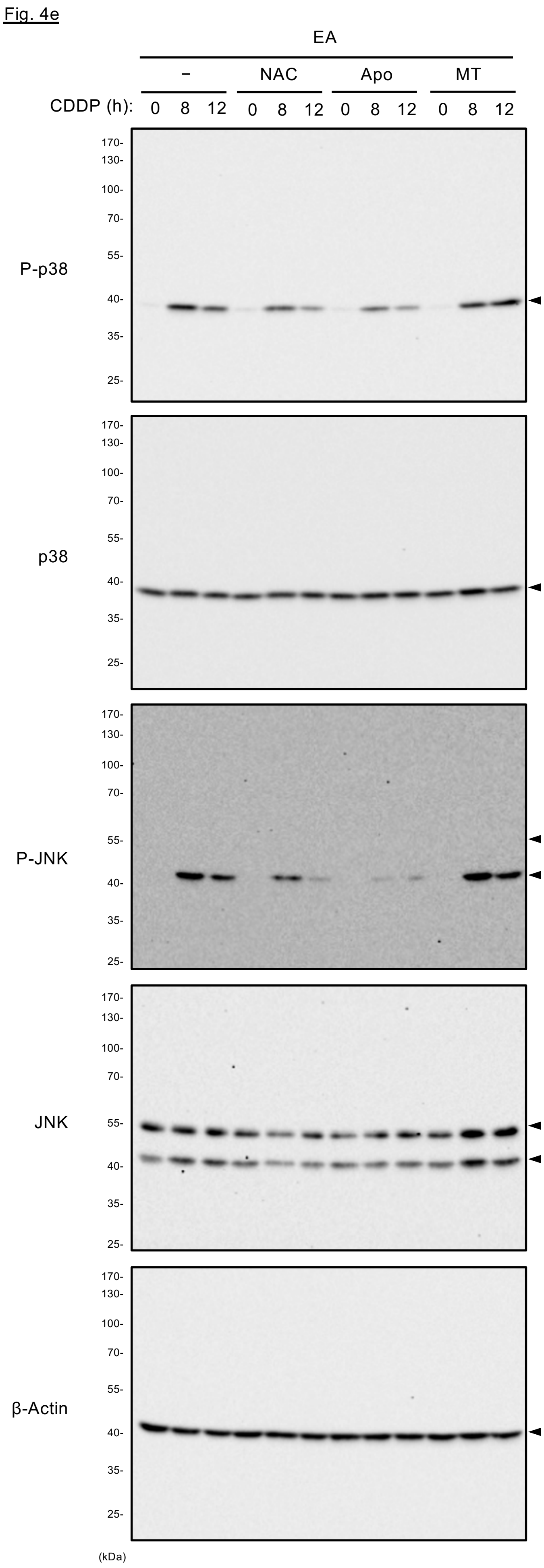

Hirata et al., Fig. S10.

j

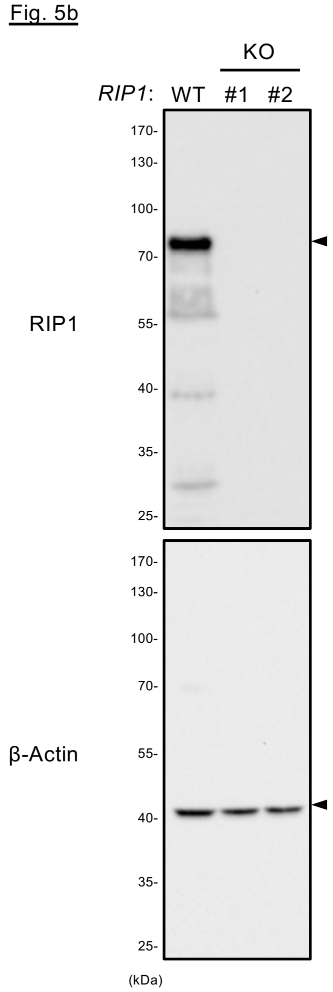

k

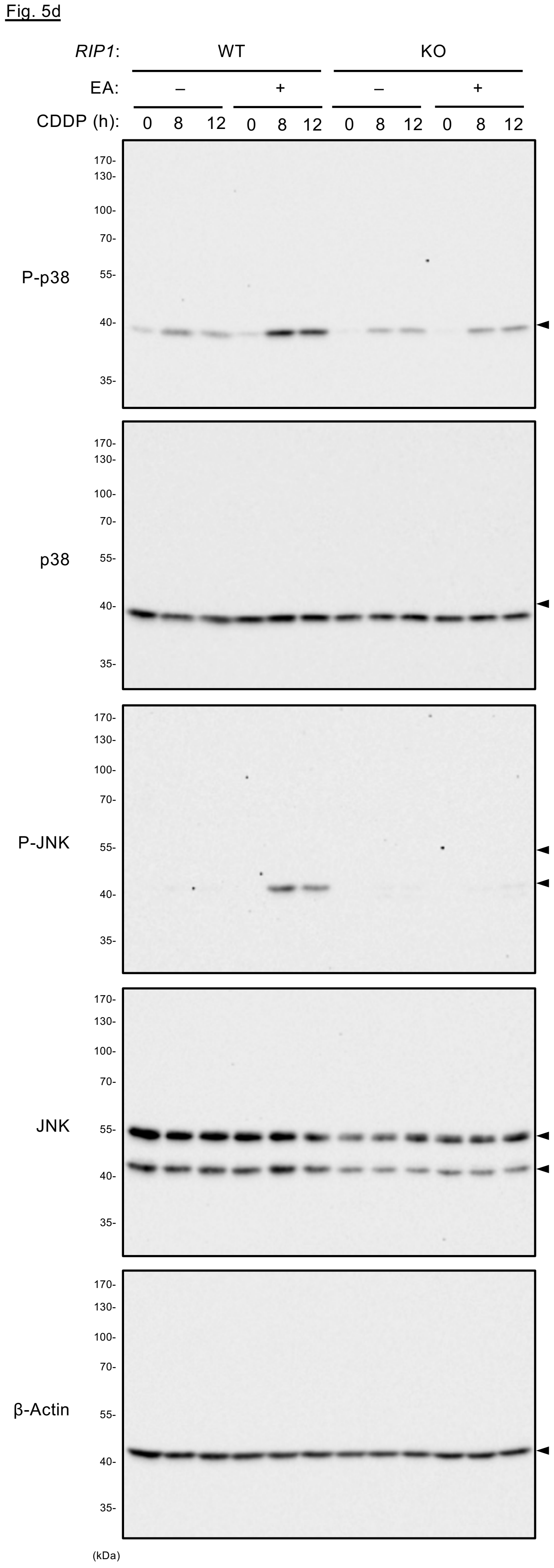

Hirata et al., Fig. S10.

l

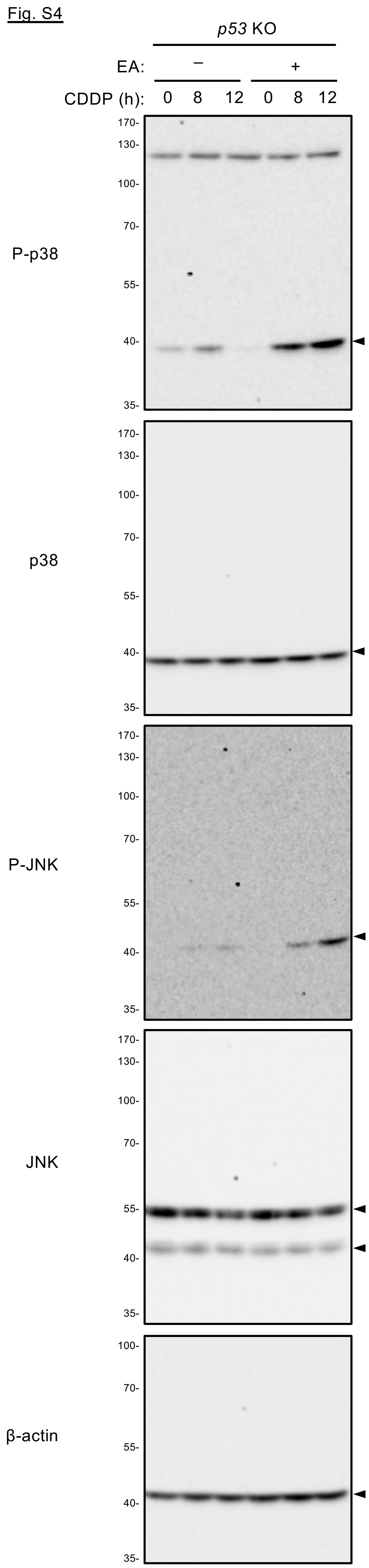

m

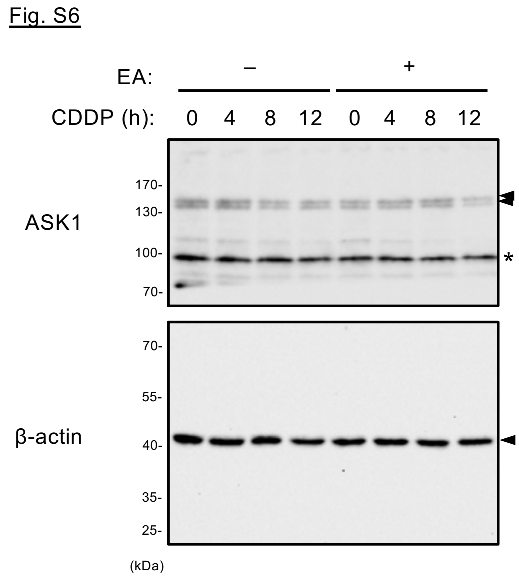

Hirata et al., Fig. S10.

n

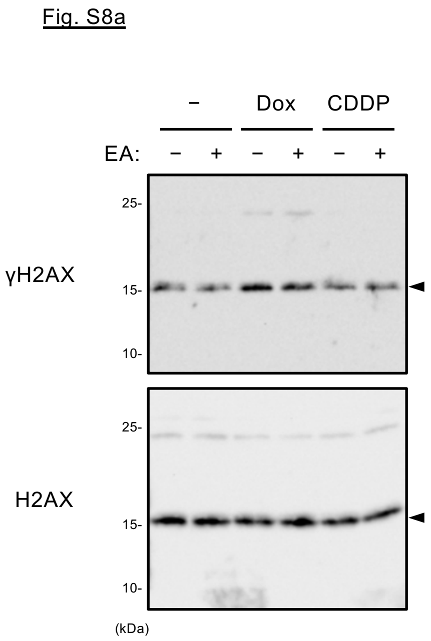

o

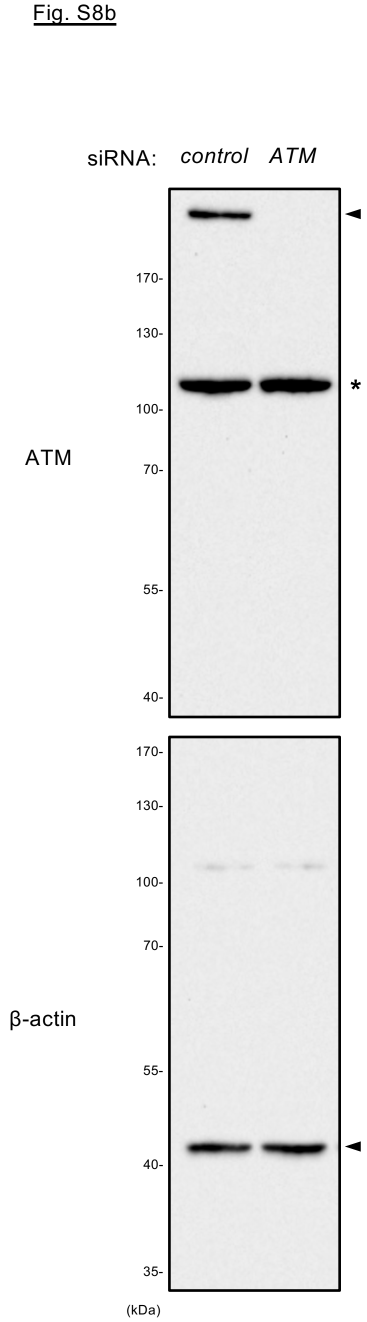

Figure S10. Full scans of immunoblot data
(a-o) Uncropped images of Fig. 1c (a), Fig. 2b (b), Fig. 2d (c), Fig. 2f (d), Fig. 3b (e), Fig. 3c (f), Fig. 3e (g), Fig. 3f (h), Fig. 4e (i), Fig. 5b (j), Fig. 5d (k), Fig. S4 (l), Fig. S6 (m), Fig. S8a (n), and Fig. S8b (o).

Hirata et al., Fig. S10.
